# Social support profiles and their association with sociodemographic and mental health characteristics among treatment-seeking adults in Arabic-speaking countries

**DOI:** 10.1371/journal.pmen.0000149

**Published:** 2025-08-11

**Authors:** Rayan El-Haj-Mohamad, Jana Stein, Nadine Stammel, Yuriy Nesterko, Birgit Wagner, Maria Böttche, Christine Knaevelsrud

**Affiliations:** 1 Clinical Psychological Intervention, Department of Education and Psychology, Freie Universität Berlin, Berlin, Germany; 2 Center ÜBERLEBEN, Berlin, Germany; 3 Department of Medical Psychology and Medical Sociology, University of Leipzig, Leipzig, Germany; 4 Clinical Psychology and Psychotherapy, Medical School Berlin, Berlin, Germany; 5 German Center for Mental Health (DZPG), partner site Berlin-Potsdam, Berlin, Germany; PLOS: Public Library of Science, UNITED KINGDOM OF GREAT BRITAIN AND NORTHERN IRELAND

## Abstract

High prevalences of mental disorders have been found among people from Arabic-speaking countries. Perceived social support has often been identified as a significant factor in the development of mental disorders, and the social environment is especially important with respect to treatment-seeking. However, the extent to which different sources of perceived social support are associated with mental health remains unclear. This cross-sectional study examined latent profiles based on perceived social support from different sources, and examined their relationship with sociodemographic characteristics, psychopathological symptom severity, and quality of life (QoL). The sample consisted of *N* = 5,977 treatment-seeking adults from different Arabic-speaking countries. Latent profile analysis was performed to identify subgroups of individuals based on perceived social support from family, friends, and significant others. Multinomial logistic regression was used to analyze predictors of profile membership. Differences between profiles regarding depressive, posttraumatic stress, somatoform symptom severity, and QoL, were examined using tests for equality of means. We identified a five-profile class-invariant unrestricted solution. Marital status, sex, age, education, and country of origin were significant predictors of profile membership. The profiles differed significantly regarding depressive, posttraumatic stress, and somatoform symptom severity, and QoL. Participants perceiving moderate to high social support from different sources indicated lower psychopathological symptom levels. The low perceived social support profile showed lower QoL compared to all other profiles (p < .05). Individuals who perceive low social support from different sources appear to show higher psychopathological symptom severity and lower QoL. Our key finding reveals that individuals with multi-source support profiles showed significantly lower psychopathological symptom severity and higher quality of life compared to those with limited support profiles. Social support perception varies systematically between participants from different Arabic-speaking countries, with individuals from more stable countries reporting higher perceived support. Therefore, clinical interventions should integrate family members and community networks as integral components to enhance therapeutic outcomes. At a systemic level, policy initiatives should focus on strengthening societal-level support infrastructure, with approaches tailored to the local political and economic contexts of the target populations. Enhancing perceived social support must be prioritized not only within psychotherapy but also through diverse societal levels, to promote sustained mental health in Arabic-speaking countries.

## Introduction

There are 22 Arabic-speaking countries, encompassing Algeria, Bahrain, Comoros, Djibouti, Egypt, Iraq, Jordan, Kuwait, Lebanon, Libya, Mauritania, Morocco, Oman, Palestine, Qatar, Saudi Arabia, Somalia, Sudan, Syria, Tunisia, United Arab Emirates, and Yemen. These countries represent diverse and heterogeneous groups. However, there are also commonalities like the shared Arabic language even though there are different dialects. In addition, there is religious diversity between, but also within countries, but the majority of Arabic-speaking countries are characterized by one religion, Islam [1], and rather collectivistic values [2]. Although research on mental health in Arabic-speaking samples is limited [3], the available studies suggest high rates of mental disorders, e.g., depression, anxiety, and posttraumatic stress disorder, in various samples from different Arabic-speaking countries, often associated with violent conflicts, economic crises, or war [4–10]. However, access to mental health care remains challenging, especially in Southwest Asia and North Africa, where there is a reported lack of psychological and psychosocial support [11]. Against this backdrop, it is crucial to gain an understanding of the factors influencing access to and utilization of mental health care. In this regard, the social context [12], and especially social barriers, are highly important in terms of treatment-seeking behavior [13]. The response of family and friends to an individual’s mental health problems influences the individual’s condition, and given that family and friends often represent the individual’s primary support network, their ability to be accepting and understanding is crucial [14]. The meaning and value of one’s social environment is of special importance in Arabic-speaking countries. Here, family and social networks serve as essential support systems, particularly in conflict-affected regions where there is no official infrastructure for social security [15]. Explanatory models have described that social support acts as a resource that mitigates the effects of stress and enables a person to cope more effectively with stressful situations [16]. Social support has been identified as a key predictor of mental health [17] and as an important factor in the development and maintenance of mental illness [18–23] being associated with different mental health symptoms [24]. Indeed, longitudinal research has illustrated that people with low levels of social support are twice as likely, for example, to develop depression as people with high levels of social support [25]. However, the relationship between social support and mental health appears to be bidirectional. While social support influences mental health outcomes, mental health status can also affect how individuals perceive available social support [18–20].

In this context, it is important to distinguish between received and perceived social support [26], with the former referring to real and actual support efforts and the latter describing the support that individuals subjectively consider to be available in their social network [26,27]. Studies have revealed that perceived social support is more strongly related to mental health compared to received social support [28–30]. The utilization of social support has previously been identified as a coping strategy in studies from conflict-affected countries in Southwest Asia [31] and among Arabic people with chronic conditions [32]. Moreover, research indicates that different sources of social support exert a differential impact on mental health [33–35]. For instance, a study with Lebanese students found that only perceived social support from family was a significant predictor of life satisfaction, and interestingly, social support from a significant other, but not from family or friends, significantly predicted positive affect [36]. In a survey of medical students in Egypt, speaking to friends or family members about mental health problems was the most likely help-seeking behavior after searching the internet [37]. Social support from friends and family has been found to be a particular protective factor that reduces the likelihood of developing depression and is negatively associated with depressive symptoms [33,38,39]. For example, a Saudi Arabian study revealed a significant association between perceived social support and depression, especially in the case of high levels of perceived social support from family or significant others [40]. Also in times of political conflict and war, social support networks play a vital role in fostering community resilience and family stability, thereby contributing to both collective life and wellbeing [15,31,41]. However, as the majority of previous studies used a variable-centered approach when trying to explain the relationship between variables in a population, the research to date may have largely overlooked differences in perceived social support between heterogeneous groups. Recently, research has begun to take an individual-based approach, such as latent profile analysis (LPA), with the aim of identifying subgroups (so-called profiles) of similar individuals within a population. This enables the investigation of predictors, correlates, or outcomes, and should lead to a better understanding of associations within and differences between these subpopulations [42]. Interested in different classes of posttraumatic stress symptoms, Stein et al. identified perceived social support as a consistent predictor of class membership [43]. Research applying such approaches has revealed that different social support profiles are differently related to mental health. For example, Li et al. [44] identified five different social support profiles, with different impacts on mental health during the COVID-19 pandemic. Kelly and Malecki [45] identified five profiles in a sample of adolescents, and found that family support has a strong impact on depressive symptoms. Burholt et al. [46] found four perceived social support profiles in a sample of older migrants, with the analyses suggesting that people with greater support from family experience higher well-being. In addition to investigating patterns of profiles, the authors used this individual-based approach to examine factors that are associated with the individual profiles. For instance, ethnicity was identified as an important predictor of profile membership, with participants’ social networks differing depending on their reported ethnicity [46]. Moreover, females were more likely to belong to profiles with higher perceived social support, which is also consistent with meta-analytic findings indicating a positive association between mental health and perceived social support in females, with a relatively high effect size [17]. Furthermore, age seems to influence profile membership: While perceived social support was found to decline across age groups during the pandemic crisis, the levels from different sources of perceived social support differed between age groups [44]. In addition, studies have illustrated that being married is associated with higher perceived social support [47,48].

To the best of our knowledge, no previous study has used an individual-based approach in Arabic-speaking countries in order to obtain a better understanding of specific perceived social support profiles in a treatment-seeking sample. As part of an internet-based psychotherapy service for Arabic-speaking participants in Arabic-speaking countries, we analyzed data from individuals who registered for this treatment in a secondary analysis.

Participants were recruited via social media, the project’s website and blog, and social networking sites. This recruitment approach typically attracts individuals actively seeking mental health support, which may result in specific demographic patterns that should be considered when interpreting findings. We hypothesize that perceived social support is significantly associated with mental health, regardless of the specific source of social support. Using an individual-based approach, the goal of the present study was to move away from the classic distinction of social support according to its amount (i.e., low, moderate, and high social support), by identifying more specific subgroups and thus achieve a better understanding of the construct. A second aim was to determine sociodemographic characteristics that predict profile membership. Third, we aimed to analyze whether profile membership was associated with psychopathological symptom severity, and hypothesized that people who perceive higher social support from different sources would report fewer psychopathological symptoms and higher quality of life.

## Methods

### Procedure and inclusion criteria

The present study is part of an internet-based therapist-assisted psychological intervention (Ilajnafsy) for people living in Arabic-speaking countries suffering from depression and/or posttraumatic stress disorder (PTSD). After registration and providing written online informed consent, interested participants undergo a two-step diagnostic process (self-assisted screening and standardized clinical interview) in order to screen for eligibility. At the beginning of the registration process, participants are informed about the voluntary, anonymous, and research-based nature of the intervention and that no financial compensation is provided. The Freie Universität Berlin Ethics Committee approved the study (185/2018).

For the present study, only cross-sectional baseline data assessed during the screening were used, i.e., prior to the clinical interview and the decision regarding eligibility for the treatment. However, our sample consists of treatment-seeking individuals who registered for an internet-based psychological intervention, reporting clinically significant levels of depressive symptoms (PHQ-9 score **M* *= 18.54, indicating moderately severe to severe depression) and other mental health concerns. For inclusion in the current analyses, participants had to be 1) aged 18 years or older and 2) able to speak, read, and write Arabic (inclusion criteria for eligibility for the intervention differ). As the intervention is available for Arabic-speaking people in general, in the present study, we decided to only include participants from those countries that represent at least 3% of the overall intervention sample (i.e., N = 8,840) to ensure meaningful statistical analyses in terms of recent country of residence. Given that the literature indicates different influences on mental health between people from different countries, country of origin was used as a covariate in the analyses. We also excluded individuals who identified as refugees due to their special vulnerable situation and its potential influence on mental health and social support.

### Sample

From February 2021 and May 2022, 8,840 participants registered to participate in the intervention and were recruited via social media, the project’s website and blog, and social and local networking sites. Of these, 1,800 were identified as refugees and were excluded from the present data pool. Thirty-four participants were younger than 18 years and 1,018 were excluded due to the country representation of under 3%. Eleven participants were identified as outliers according to Mahalanobis distance and therefore excluded from the final analyses. Overall, the present study included N = 5,977 Arabic-speaking treatment-seeking adults from the following countries: Egypt (38.2%), Saudi Arabia (31.7%), Jordan (8.6%), Morocco (7%), Algeria (5.2%), Iraq (5.4%), and the United Arab Emirates (3.9%). Participants’ age ranged between 18 and 85 years (M = 23.54, SD = 6.37). The majority of participants were female (77%), educated (high school-leaving qualifications, 53.3% or university degree, 35.5%), and not in a relationship or marriage (71.8%). Most of the participants had no children (86%), while 4.3% had one child, 4.7% had two children, and 4.5% had more than four children. Most of the participants reported having more than one sibling (two: 20.1%, three: 19.1%, four: 15.4%, five: 10.9%, six: 7%; more than six: 17.6%). Only a small proportion of participants had either no siblings (2.3%) or one sibling (9.8%).

### Measures

For the assessment of social support, depressive, somatoform, and posttraumatic stress symptom severity, and quality of life, participants completed standardized self-assessment questionnaires online. Measures that were not available in standard Arabic at the time of planning the study were translated using forward-backward translation. Additionally, sociodemographic variables such as gender, age, country of origin, marital status, education, number of children, and siblings were surveyed.

#### Social support.

Perceived social support was measured using the validated Arabic version [49] of the Multidimensional Scale of Perceived Social Support (MSPSS; [50]. The 12-item scale encompasses three subscales covering the dimensions of social support from friends (items 6, 7, 9, and 12), family (items 3, 4, 8, and 11), and a significant other (items 1, 2, 5, 10), with items rated on a 7-point Likert scale ranging from 1 (strongly disagree) to 7 (strongly agree). For each of the three subscales, a mean score was calculated by summing the items of each dimension and dividing the total by four. The scale has shown good psychometric properties in Arabic-speaking samples [49]. The reliability in the present sample was excellent, at *α* = .90.

#### Depressive symptoms.

Depressive symptoms in the past two weeks were assessed using a validated Arabic version [51] of the Patient Health Questionnaire - 9 (PHQ-9; 46). The scale measures the presence and severity of depressive symptoms using nine items rated on a 4-point Likert scale ranging from 0 (not at all) to 3 (almost every day), with sum scores of 5–9, 10–14, 15–19, and ≥ 20 indicating mild, moderate, moderately severe, and severe levels of depression, respectively [52]. The PHQ-9 has shown good psychometric properties in Arabic-speaking samples [51,53]. The reliability in the present sample was good, at *α* = .83.

#### Somatoform symptoms.

Somatoform symptoms in the past four weeks were assessed using a translated version of the Patient Health Questionnaire **-** 15 (PHQ-15; [54]), which has shown good psychometric properties, also in Arabic-speaking populations [55]. The scale measures somatic symptoms using 15 items rated on a 3-point scale ranging from 0 (not impaired) to 2 (strongly impaired), with sum scores of 5–9, 10–14, and ≥ 15 indicating mild, moderate, and severe somatoform symptom severity, respectively. Good reliability and validity have been found in Arabic-speaking samples [51,56]. Internal consistency in the present sample was also good, at *α* = .82.

#### Posttraumatic stress symptoms.

The self-report Posttraumatic Stress Disorder Checklist for DSM-5 (PCL-5) was used to assess posttraumatic stress symptom severity [57]. The scale was self-translated into Arabic and consists of 20 items rated on a 5-point Likert scale ranging from 0 (not at all) to 4 (extremely). A sum score was calculated, with higher sum scores indicating greater symptom severity. The PCL-5 has shown good psychometric properties in Arabic-speaking samples [58,59]. In the present sample, internal consistency was excellent, at *α* = .91.

#### Quality of life.

Participants’ psychological, physical, social, and environmental quality of life was assessed using a self-translated version of the European Health Interview Survey 8-Item Index (EUROHIS-QOL-8;54), which consists of eight items rated on a 5-point Likert scale ranging from 1 to 5 (different response formats). A sum score was calculated, with higher scores indicating higher quality of life. Although there is no psychometrically validated Arabic version of the EUROHIS-QOL, international studies have demonstrated acceptable to good cross-cultural performance [60,61]. In the present study, internal consistency was acceptable, at *α* = .76.

### Statistical analysis

Given that outliers reduce the power of statistical tests and increase error variance [62], we first identified outliers using Mahalanobis distance, a measure of distance that considers the intercorrelations among the variables with multivariate data and allows for the detection of outliers [62]. Second, the original three-factor structure of the MSPSS (i.e., family, friends, and significant others) was examined using confirmatory factor analysis (CFA) to control for the factor structure in an Arabic-speaking sample. To assess model fit, the following criteria were considered: The standardized root mean square residual (SRMR) with values lower than.08, the parsimony-adjusted root mean square error of approximation (RMSEA, values closer to 0 represent a good fit), and the comparative fit index (CFI), which compares the model fit to an independent or null model, with values > .90 representing a good fit [63–65]. The CFA results supported the three-factor structure of the MSPSS, indicating the original three subscales of family, friends, and significant others, χ2 (39) = 1597.322, p < .001, CFI = .968, RMSEA = .071 (CI = .068 -.074), SRMR = .035.

Third, a latent profile analysis (LPA) using *M*Plus (Version 8.7) with maximum likelihood estimation was performed to identify the number of subgroups within the sample based on individual differences in perceived social support from family, friends, and significant others. As recommended by Masyn [66], different models of variance-covariance specifications were compared to find the best-fitting model. We began by examining the most restrictive form of LPA model, the *class-invariant, diagonal(a).* Here, covariance is not permitted within classes and variances are constrained to be equal across classes. The class-*varying diagonal (b)* specification allows for a different variation in each profile across the classes, while covariance within classes is fixed at zero. The third model, the *class-invariant, unrestricted (c)*, allows covariance within classes, and variances and covariances are estimated to be equal across classes. The *class-varying, unrestricted (d)* structure allows for covariance within classes, and covariances and variances are allowed to be different across classes [66]. Models for which the best log-likelihood value could not be replicated were not considered [66]. To decide on the optimal model solution, we considered the following statistical criteria [67]: Profiles were compared using the Akaike information criterion (AIC), the Bayesian information criterion (BIC), and the sample-size-adjusted Bayesian information criterion (SABIC), where lower values indicate a better fit. Other decision parameters were a significant Lo-Mendell-Rubin likelihood ratio test (LMR-LRT), bootstrap likelihood ratio test (BLRT), and higher entropy values [68]. Sample size was considered, as classes with a small sample size may indicate that too many classes have been extracted [67,68]. Furthermore, theoretical parsimony and the interpretability of the content were taken into account [68–70]. Lastly, as recommended by Masyn (60) and Johnson [71], the BIC values were plotted over the profiles to locate the point of largest decrease.

To assess predictors of profile membership, we applied the three-step approach for multinomial logistic regression: 1) building classes based on a set of variables, 2) assigning participants to classes, 3) investigating the relationship between class membership and external variables), which takes the inaccuracy of class assignment into account [72]. Predictors included sociodemographic characteristics (age, sex, education, number of children and siblings, country of origin, marriage/relationship status). For better interpretation, the variable assessing relationship status was dichotomized: Individuals in a relationship or marriage were coded as (1), indicating a relationship, and individuals who were single, widowed, or divorced were coded as (0), indicating no relationship. Finally, to test for equality of means between profiles regarding the severity of depressive, somatoform, and posttraumatic stress symptoms as well as quality of life, the three-step procedure was used [73,74]. This approach enables the inaccuracy of class assignments to be taken into consideration. Analyses were performed using *M*Plus Version 8.7.

## Results

### Sample characteristics

Overall, participants reported a medium level of perceived social support (M = 3.29, SD = 1.43), with the highest perceived social support from significant others (M = 3.72, SD = 1.95), followed by friends (M = 3.29, SD = 1.80) and family (M = 3.03, SD = 1.69). Table 1 illustrates the descriptive characteristics of perceived social support from family, friends, and significant others as well as symptom severity for all mental health measures and QoL.

**Table 1:** Descriptive characteristics of social support, psychopathology, and quality of life (N = 5,977).

	M	SD	Range
Perceived social support (MSPSS)	3.29	1.43	1-7
From family	3.03	1.69	
From friends	3.29	1.80	
From significant other	3.72	1.95	
Depressive symptom severity (PHQ-9)	18.54	5.74	0 - 27
Somatoform symptom severity (PHQ-15)	15.03	5.62	0 - 30
Posttraumatic stress symptom severity (PCL-5)^*^	48.10	16.26	0 - 80
Quality of life (EUROHIS-QOL)	11.97	5.38	0 - 21

*Note*.

* N = 4,634;

M = Mean; SD = Standard deviation; PHQ-9 = Patient Health Questionnaire-9; PHQ-15 = Patient Health Questionnaire-15; PCL-5 = Posttraumatic Symptom Checklist-5 for Diagnostic and Statistical Manual of Mental Disorders-5; EUROHIS-QOL = EUROHIS Quality of Life 8-item index.

### Latent profile analysis

To identify the optimal number of profiles to fit the data, eight profiles were estimated for the *class-invariant diagonal* and *unrestricted* structure. When examining *class- varying diagonal and unrestricted* models, only three profiles were interpretable, because the best log-likelihood value could not be replicated with extremely high numbers of random starts for the profiles with more classes. Therefore, we stopped increasing profile numbers for these models. Statistical criteria and further information for different profiles and models are provided in Table 2.

**Table 2:** Goodness-of-fit information for different latent profiles (N = 5,977).

Model	No. of profiles	AIC	BIC	SABIC	Entropy	LMR-LRT p-value	BLRT p-value	Smallest sample size in a profile
Class-invariant, diagonal (a)	2	68337.916	68404.873	68373.096	.799	<.001	<.001	2962
	3	67502.147	67595.886	67551.398	.842	<.001	<.001	1103
	4	67017.035	66553.860	66617.183	.802	<.001	<.001	877
	5	66138.284	66285.589	66215.679	.796	.003	<.001	769
	6	65772.533	65946.620	65863.999	.825	<.001	<.001	237^°^
	7	65593.736	65794.606	65699.274	.815	<.001	<.001	394
	8	65326.659	65554.311	65446.269	.805	.016	<.001	251^°^
Class-varying, diagonal (b)	2	66346.498	66433.541	66392.231	.920	<.001	<.001	1277
	3	65296.686	65430.599	65367.045	.935	<.001	<.001	298^°^
Class-invariant, unrestricted(c)	2	67745.894	67832.937	67791.627	.781	<.001	<.001	2417
	3	66991.262	67105.088	67051.067	.839	<.001	<.001	1072
	4	66329.096	66469.705	66402.973	.823	<.001	<.001	611
	**5**	**65866.488**	**66033.880**	**65954.437**	**.813**	**<.001**	**<.001**	**531**
	6	65639.737	65833.912	65741.758	.813	<.001	<.001	238^°^
	7	65359.685	65580.643	65475.778	.826	<.001	<.001	496
	8	65186.713	65434.453	65316.877	.826	<.001	<.001	198^°^
Class-varying, unrestricted(d)	2	66346.498	66433.541	66392.231	.920	<.001	<.001	1277
	3	64484.567	64678.741	64586.587	.882	<.001	<.001	384

*Note.* BIC = Bayesian information criterion; SABIC = sample-size-adjusted Bayesian information criterion; LMR-LRT = Lo-Mendell-Rubin adjusted likelihood ratio test; BLRT = bootstrapped likelihood ratio test;

°  = profile with a sample size < 5%.

Most meaningful model is printed in **bold**.

Across all profiles, the statistical criteria AIC, BIC, and SABIC became smaller and the BLRT and adjusted LMR became significant when adding a profile. *Class varying diagonal* and un*restricted* models showed overall lower AIC, BIC, SABIC, and higher entropy, but only allowed for the consideration of two or three profiles with small sample sizes.

The comparison of the statistical criteria between the class-invariant diagonal and *unrestricted* models showed similar statistical criteria across the profiles, with the tendency that the unrestricted structure represented the data more accurately since the statistical parameters AIC, BIC, and SABIC were lower and the entropy was higher (Table 2).

*Class-invariant unrestricted models* were therefore preferred over class-*varying diagonal* models.

The *class-invariant unrestricted* solutions showed acceptable entropy values (>.80) for all solutions ranging from three to eight profiles, indicating clear profile separation. Several statistical and practical considerations led to the final selection of the five-profile solution: 1) The three- and four-profile solutions showed inferior model fit indices (AIC, BIC, SABIC) compared to solutions with more profiles; 2) For solutions with six and eight profiles, some subgroups comprised less than 5% of the sample, limiting practical interpretability, 3) When examining the BIC values (see S1 Fig), solutions with six, seven, and eight profiles showed no substantial improvements in model fit compared to the five-profile solution. Based on these criteria, the five-profile class-invariant unrestricted model was selected as it provided the best balance between statistical fit, practical interpretability, and profile distinction.

### Description of the profiles

**Fig 1:**
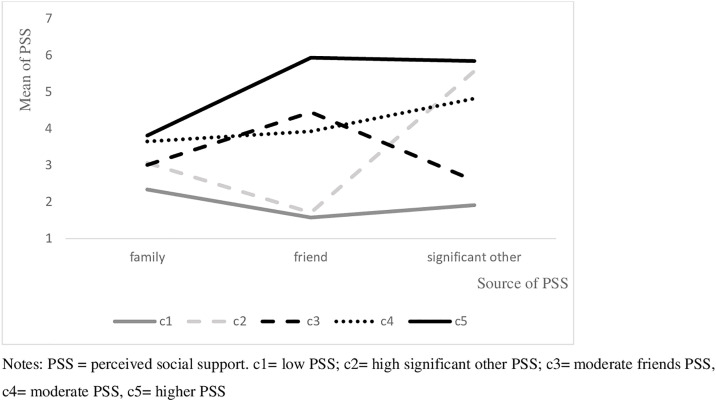
Profiles of the five-profile class-invariant unrestricted solution.

The overall best-fitting model consisted of five profiles (of model c, Table 2, Fig 1). The first profile was characterized by low perceived social support (PSS) from all sources (*low PSS*; n = 2,365; 39.56%, c1). In the second profile, perceived social support was moderate from family, low from friends, and high from significant others (*high significant other PSS*; n = 697; 11.66%, c2). The third profile seems to represent a mirror image of c2, with moderate perceived social support from family and friends and low perceived social support from significant others (*moderate friends PSS*; n = 531; 8.88%, c3). The fourth and fifth profiles also showed a mirroring pattern: While perceived social support from family was moderate in both groups, perceived social support from friends and significant others was moderate to high in the fourth profile *(moderate PSS* n = 1458; 24.34%, c4) and high in the fifth profile (*higher PSS* n = 926; 15.49%, c5). The average latent profile probabilities for the most likely latent profile membership with the five-profile solution was high, ranging between.81 and.94.

### Profile membership characteristics

Table 3 provides an overview of the demographic characteristics within the five profiles. Gender, education, age, country of origin, and number of children and siblings appear to be similarly distributed between the profiles, while relationship status, in terms of being in partnership/marriage, appears to differ between the profiles.

**Table 3:** Variable characteristics by latent profiles for the five-profile solution.

N (% of category in profile)					
Profiles	Low PSS (n = 2,365)	High significant other PSS (n = 697)	Moderate friends PSS (n = 531)	Moderate PSS (n = 1,458)	Higher PSS (n = 926)
**Female**	1,759 (74.4%)	563 (80.8%)	414 (78%)	1,088 (74.6%)	777 (83.9%)
**Education**					
No school-leaving qualifications	43 (1.8%)	20 (2.9%)	4 (0.8%)	36 (2.5%)	14 (1.5%)
Medium-track secondary school	250 (10.6%)	67 (9.6%)	34 (6.4%)	127 (8.7%)	75 (8.1%)
Higher-track secondary school	1,250 (52.9%)	365(52.4%)	309 (58.2%)	733 (50.3%)	529(57.1%)
University degree	822 (34.8%)	245(35.2%)	184 (34.7%)	562 (38.5%)	308(33.3%)
**No children**	2,089 (88.3%)	590 (87.6%)	478 (90%)	1217 (83.5%)	811(87.6%)
**Partnership/ marriage**	444 (18.8%)	339 (48.6%)	91 (17.1%)	498 (34.2%)	308(33.3%)
**Country of origin**					
Algeria	121 (5.1%)	54 (7.7%)	25 (4.7%)	74 (5.1%)	38 (4.1%)
Iraq	129 (5.5%)	33(4.7%)	24 (4.5%)	77 (5.3%)	57 (6.2%)
Jordan	196 (8.3%)	51 (7.3%)	52 (9.8%)	142 (9.7%)	74 (8.0%)
Morocco	165 (7.0%)	62 (8.9%)	21 (4.0%)	108 (7.4%)	63 (6.8%)
Saudi-Arabia^3^	709 (30.0%)	226 (32.4%)	156 (29.4%)	490 (33.6%)	313(33.8%)
United Arab Emirates^3^	92 (3.9%)	37 (5.3%)	16 (3.0%)	56 (3.8%)	35 (3.8%)
Egypt	953 (40.3%)	234 (33.6%)	237 (44.6%)	511 (35%)	346 (37.4%)
**Mean (SD)**					
**Age**	23.33 (6.3)	23.78 (6.67)	22.78 (4.8)	24.24 (6.78)	23.24 (6.34)
**Siblings**	4.03 (2.83)	4.13 (3.02)	3.91 (2.53)	4.2 (2.89)	3.98 (2.78)

Notes: N = Number of participants, SD = Standard deviation.

We further conducted a multinomial logistic regression analysis with demographic variables as predictors and profile membership as the dependent variable (Table 4). The low PSS profile was used as the reference category, as this profile includes the most participants, the lowest social support from all sources, and contrasted best with the other profiles. Five participants were excluded from this analysis due to missing data on demographic variables. Compared to the low PSS profile (c1), the likelihood of membership of the high significant other PSS profile (c2) was significantly higher for females, for participants in a relationship, participants with children, and participants who originated from Algeria, Morocco, Saudi Arabia, and the United Arab Emirates as compared to Egypt.

**Table 4:** Results of the multinomial logistic regression predicting profile membership (N = 5,972) with the overall low PSS profile as reference group.

Profiles	High significant other PSS			Moderate friends PSS			Moderate PSS			Higher PSS		
Variables	B	SE	p	B	SE	p	B	SE	p	B	SE	p
**Sex** ^1^	.29	.14	**<.05**	.24	.15	.12	-.11	.10	.28	.62	.13	**<.01**
**Education** ^2^	-.09	.06	.11	.14	.06	**<.05**	.01	.04	.94	-.02	.05	.72
**Children**	-.19	.09	**<.05**	-.01	.03	.61	-.03	.04	.42	-.08	.15	.58
**Siblings**	-.01	.02	.55	-.01	.02	.91	-.01	.02	.68	-.03	.02	.11
**Age**	.01	.01	.90	-.03	.01	**<.05**	.01	.01	.22	-.01	.01	.66
**Partnership/marriage** ^3^	1.79	.12	**<.01**	-.27	.20	.18	.96	.10	**<.01**	.95	.12	**<.01**
**Algeria** ^3^	.75	.21	**<.01**	-.37	.32	.25	.30	.2	.14	-.16	.23	.48
**Iraq** ^3^	.03	.27	.91	-.43	.29	.14	.22	.19	.25	.21	.19	.27
**Jordan** ^3^	.12	.21	.56	.06	.21	.79	.41	.16	**<.05**	.04	.18	.82
**Morocco** ^3^	.52	.20	**<.01**	-.94	.36	**<.01**	.36	.17	**<.05**	.04	.19	.83
**Saudi Arabia** ^3^	.37	.15	**<.01**	-.25	.18	.16	.43	.12	**<.01**	.26	.12	**<.05**
**United Arab Emirates** ^3^	.55	.25	**<.05**	-.49	.37	.19	.27	.22	.23	.04	.24	.88

*Note*.

^1^male = 0, female = 1;

^2^no school-leaving qualifications = 0, medium-track secondary school = 1, higher-track secondary school = 2, university degree = 3;

^3^no = 0, yes = 1.

B = regression coefficient estimates; SE = standard error; Country was dummy-coded with Egypt as the reference group.

Compared to the low PSS profile (c1), the likelihood of membership of the moderate friends PSS profile (c3) was significantly higher for younger participants and for participants with higher educational attainment, but significantly lower for participants who originated from Morocco as compared to Egypt.

Compared to the low PSS profile (c1), the likelihood of membership of the moderate PSS profile (c4) was significantly higher for participants in a relationship and for participants who originated from Jordan, Morocco, and Saudi Arabia as compared to Egypt. Finally, compared to the low PSS profile (c1), the likelihood of membership of the higher PSS profile (c5) was significantly higher for females, for participants in a relationship, and for participants who originated from Saudi Arabia as compared to Egypt.

### Mean differences in symptom severity and quality of life between profiles

The results of the overall test for equality of means indicated significant group differences in depressive symptom severity (*χ*^*2*^(4) = 210.08, *p* < .001), somatoform symptom severity (*χ*^*2*^(4) = 24.23, *p* < .001), posttraumatic stress symptom severity (*χ*^*2*^(4) = 84.45, *p* < .001), and quality of life (χ^2^(4) = 497.38 *p* < .001).

Table 5 shows the pairwise comparison between all profiles. Compared to the low PSS profile, participants in all other profiles reported significantly lower depressive symptoms, except for c3 (moderate friends PSS profile), which did not significantly differ.

**Table 5:** Means, standard error and pairwise test for equality of means for symptom severity, with comparison to the overall low perceived social support profile (N = 5,977)^+^.

	Low PSS (c1)	High significant other PSS (c2)	Moderate friends PSS (c3)	Moderate PSS (c4)	Higher PSS (c5)	Significant differences between profiles^*^
Depressive Symptoms M (SE)	19.76 (.12)	18.69 (.25)	19.35 (.28)	16.85 (.20)	17.46 (.23)	c1 ~ c2, c4, c5 c2 ~ c5 c3 ~ c5 c4 ~ c2
Somatoform Symptoms M (SE)	15.25 (.13)	15.48 (.25)	15.11 (.29)	14.22 (.19)	15.33 (.21)	c1 ~ c4 c2 ~ c4 c4 ~ c5
Posttraumatic stress symptoms M (SE)	50.29 (.40)	49.90 (.76)	49.05 (1.01)	44.58 (.63)	45.73 (.71)	c1 ~ c4, c5 c2 ~ c4, c5 c3 ~ c5
Quality of Life M (SE)	10.15 (.11)	12.13 (.24)	11.73 (.28)	13.43 (.71)	14.26 (.21)	c1 ~ c2, c3, c4, c5 c2 ~ c5, c4 c3 ~ c5 c4 ~ c5

Note: M = Mean; SE = Standard error; PSS = Perceived Social Support;

* pairwise test for equality of means;

+ Analysis for equality of means was conducted with N = 5,977 participants, except for the variable posttraumatic stress symptom severity (n = 4,634).

Compared to the low PSS profile (c1), participants in the moderate PSS profile (c4) reported significantly lower somatoform symptoms. There were no significant differences between the low PSS profile (c1) and the other profiles (c2, c3, c5) regarding somatoform symptom severity.

Compared to the low PSS profile (c1), participants in the moderate PSS (c4) and higher PSS profiles (c5) reported significantly lower posttraumatic stress symptom severity, whereas participants in the moderate friends PSS (c3) and high significant other PSS profiles (c2) did not significantly differ from those in the low PSS profile (c1).

The comparisons between the low PSS profile (c1) and all other profiles revealed significant differences regarding quality of life.

## Discussion

The first aim of this study was to examine profiles of social support from family, friends, and significant others in adults from different Arabic-speaking countries. Second, we investigated the association between sociodemographic characteristics and profile membership, and third, we examined differences between profiles regarding depressive, somatoform, and posttraumatic stress symptom severity as well as quality of life.

The latent profile analysis resulted in a five-profile class-invariant unrestricted solution based on perceived social support from different sources, with the following profiles: low PSS (c1); high significant other PSS (c2), moderate friends PSS (c3), moderate PSS (c4), and higher PSS (c5). A five-profile solution is consistent with other studies that analyzed profiles of perceived social support from different sources [44–46]. While the previous studies did not consider different restrictions when deciding on the best-fitting model, as suggested by Masyn et al. [66], we were able to identify overall similar profiles, with a lower, a moderate, and a higher PSS profile. Moreover, we additionally identified a profile with moderate to high PSS from friends (c3) and a profile with high PSS from significant others but low PSS from friends and moderate PSS from family (c2).

Overall, the largest proportion of participants (n = 2,365; 39.57%) in our study belonged to the low PSS profile. This profile is characterized by the highest depressive and PTSD symptom severity and the lowest QoL. Interestingly a previous conducted research in Germany, analyzing help-seeking classes in untreated populations identified a class characterized by participants that mostly sought help from mental health professionals and less from friends or family and was signified by participants reporting lower well-being and more severe depressive symptoms [75]. Given that we assessed perceived social support, it remains unclear whether these individuals actually receive less social support or whether they merely perceive this to be the case, as research has indicated that people with mental illnesses generally perceive lower social support [18–20].

The second largest profile was the moderate PSS, containing 24.39% of participants (n = 1,458), followed by higher PSS (n = 926; 15.49%), high significant other PSS (n = 697; 11.66%), and moderate friends PSS (n = 531; 8.88%).

Although previous studies on the family as the most important source of social support in samples from Arabic-speaking countries have yielded divergent findings [36,39,40], the present findings illustrate that family appears to play a moderate role across all profiles, with the exception of the low PSS profile (c1). However, none of the profiles showed high perceived social support from the family. One reason for this might lie in a fear of stigmatization. Indeed, a review by Zolezzi et al. [76] examining stigmatization of mental illness in Arabic samples illustrated that mental health-related stigma is negatively related to the reputation of the entire family. However, perceived social support is crucial, because disturbances in relationships might be related to poor mental health [77]. A study in Egypt illustrated that familial involvement positively impacted recovery in individuals with mental illness [78].

In line with previous studies with Arabic-speaking participants, which reported higher perceived social support among married participants [47], and underpinning the importance of spouses/partners [32], we found that marital status was significantly associated with membership of most of the profiles: Participants who were married or in a relationship were more likely to belong to the moderate PSS (c4), higher PSS (c5), or high significant other PSS profile (c2) compared to the low PSS profile (c1).

In the current study, females were more likely to belong to the higher PSS (c5) and high significant other PSS profiles (c2) compared to the low PSS profile (c1). A recent review analyzing mental health help-seeking in Arab samples found that women reported higher help-seeking intentions and more positive attitudes towards seeking help than did men [79]. This active help-seeking behavior might also influence the perception of social support and might explain the distribution in the current study. Moreover, this finding emphasizes the greater openness of women in general to seek external help and the lower level of perceived stigmatization of mental health problems in women [80–82], as men are exposed to a stronger cultural stigma than women and may associate seeking help for their mental health as a weakness and diminishment of their masculinity [83].

Younger individuals with a higher educational level were more likely to belong to the moderate friends PSS profile (c3) compared to the low PSS profile (c1). Due to the sample characteristics in terms of age (young adults) and education (current highest educational attainment was high school), we assume that most of these individuals might have been university students at the time of assessment, representing a period of life when friends play a particularly important role and positive associations between perceived social support from friends and mental health are strong [33,84].

Country of origin was significantly related to profile membership: Compared to participants from Egypt, participants from Saudi Arabia were more likely to belong to the high significant other PSS (c2), moderate PSS (c4), and higher PSS (c5) profiles than to the low PSS profile (c1). Moreover, participants from Jordan and Morocco showed a higher likelihood of belonging to the moderate PSS profile (c4) than people from Egypt, with the low PSS profile as the reference category (c1). One explanation of these differences might be linked to socioeconomic and political factors. Egypt is considered to be one of the Arabic-speaking countries with fewer resources, a lower-middle income, and a high unemployment rate [11,85,86]. Furthermore, it is characterized by political conflicts and thus reflects a financially and politically more unstable country. In contrast, Jordan is considered as an upper-middle income country and both Jordan and Morocco tend to be politically rather stable [86]. Saudi Arabia is not only rich in resources but is also characterized by a low unemployment rate and is also politically stable [86]. Better access to health facilities and thus a higher health literacy might influence perceived social support. In a report examining the development of mental health services and progress in Arabic-speaking countries, Saudi Arabia scored the highest in the domain of accessibility of mental health services [87], which might in turn lead to more active help-seeking behavior and therefore also to more perceived social support. Nevertheless, the interpretation of these differences in perceived social support profiles between countries remains challenging. Harb analyzed value differences between Arabic-speaking countries and found substantial variations between countries, with differences on various dimensions (e.g., uncertainty, power, [88]. However, the author highlighted that some findings appeared counterintuitive and may reflect sampling biases. The differences in values between Arabic-speaking countries have not been sufficiently investigated and more systematic bottom-up research is needed to better understand value structures in Arabic-speaking countries [88]. This limitation in understanding differences between countries also applies to the current findings on social support differences and the underlying value structures and attitudes that might explain these differences need further investigation.

Lastly, we examined differences in depressive, somatoform, and posttraumatic stress symptom severity as well as quality of life across profiles. The results illustrated that participants who perceived generally low social support showed higher symptom severity than those who perceived more social support. Depressive symptom severity was significantly higher in the low PSS profile (c1) compared to the moderate PSS (c4), higher PSS (c5), and high significant other PSS (c2) profiles. This corresponds to several reviews with Western samples [18,22], mixed samples [20], and Southwest Asian samples [32,89], which likewise indicated associations between high depressive severity and low perceived social support. Overall somatoform symptom severity was comparable across all profiles (mean ranging from 14.22 – 15.48) and was classified as severe in all profiles (mean score above the cut-off of 15 on the PHQ-15, 81). We did not find differences in somatoform symptoms when comparing low PSS with the other profiles (except for the moderate profile), which might be attributable to the comparably high severity across all profiles. Regarding posttraumatic stress symptom severity, the low PSS profile (c1) differed significantly from the moderate PSS profile (c4) and from the higher PSS profile (c5). Participants in profiles with higher perceived social support from different sources showed lower posttraumatic stress symptom severity, corresponding to recent meta-analytic findings that indicated negative moderate reciprocal associations between social support and PTSD [23,35]. Our results additionally shed light on the source of social support, revealing that different sources (i.e., family, friends, and significant others) have a differential impact on posttraumatic stress symptom severity. Comparisons between the low PSS profile (c1) and all other profiles (c2-c5) indicated significant differences with regard to quality of life, with the lowest values in the low PSS profile (c1). Previous studies likewise demonstrated that low perceived social support is associated with lower QoL [90–93]. In this regard, the perception of social support from one source might be sufficient for a higher QoL, but QoL is highest when social support is perceived from different sources.

## Limitations

Several limitations of the present study need to be acknowledged. First, the sample consists of treatment-seeking individuals, who represent a more mentally burdened group compared to the general population, which might have an influence on the perception of PSS. Second, in terms of sample characteristics, the participants were mainly educated, single, female adults. Although, these characteristics are typical for treatment-seeking samples [94], this sample characteristic should be considered when interpreting our results and attempting to generalize the findings. Third, the sample comprises individuals from seven Arabic-speaking countries (Egypt, Saudi Arabia, Jordan, Morocco, Algeria, Iraq, and the United Arab Emirates). Accordingly, conclusions can only be drawn regarding these specific countries and not regarding Arabic-speaking countries in general. Fourth, regarding our methodological approach, we used a binary relationship status coding where individuals who were single, divorced, or widowed were categorized as ‘not in a relationship,’ while married individuals and those in partnerships were categorized as ‘in a relationship.’ While this represents research practice and facilitates clearer interpretation of predictors, future research with sufficient sample sizes should investigate whether meaningful differences exist between subgroups within each category. Lastly, we assessed general perceived social support separated into different sources of support (i.e., family, friends, significant other). Support might also be assessed in terms of actual received support or with respect to information-based (e.g., advice), emotional (e.g., listening), or instrumental (e.g., financial aid) social support. As such, the findings are based only on assumptions regarding self-perceived PSS, while different types of social support may show different associations with mental health [18] and participants might perceive social support differently in relation to the topic (e.g., mental health problems, financial problem).

## Conclusion

A focus on risk and protective factors for mental health problems is especially important in countries with a lack of health professionals and high prevalence rates of mental disorders. One such factor is social support, which appears to facilitate active help-seeking behavior and promote mental health. While previous research has demonstrated the impact of perceived social support, further studies are required to gain a better understanding of the mechanisms of perceived social support and of social support in different regions of the world. As a first step in this direction, we found five profiles of perceived social support in participants from seven Arabic-speaking countries and examined their associations with psychopathological symptom severity and quality of life. In general, individuals who perceived social support from multiple sources showed lower symptom severity and higher quality of life. Future research needs to examine different forms of social support in greater detail, as previous findings have indicated that a lack of emotional support plays a greater role in the development of mental disorders like depression compared to a lack of instrumental support [18]. In terms of practical implications, our results suggest that the factor of social support should be addressed explicitly in counseling and/or psychotherapy with the aim of either improving or setting up support structures and fostering the individual’s perception of social support. This can be operationalized by integrating supportive individuals into the therapy itself (e.g., friends or family members) or by offering community-based approaches in which social networks and social connections are also (re)activated or newly established. The involvement of family members could be particularly beneficial when working with Arabic-speaking clients and is an aspect that should be considered in the development of treatment programs and taken into account in future research [95,96]. Finally, the present findings illustrate that social support is perceived differently between Arabic-speaking countries, insofar as individuals from more financially and politically stable countries appear to perceive more social support. Importantly, our study emphasizes the diversity of individuals from different Arabic-speaking countries, who are frequently treated as one homogeneous group [97].

## Supporting information

S1 Fig Plotted Bayesian information criterion. (TIFF)
